# Covalent Surface Functionalization of Bovine Serum Albumin to Magnesium Surface to Provide Robust Corrosion Inhibition and Enhance In Vitro Osteo-Inductivity

**DOI:** 10.3390/polym12020439

**Published:** 2020-02-13

**Authors:** Seo Yeon Lee, Sita Shrestha, Bishnu Kumar Shrestha, Chan Hee Park, Cheol Sang Kim

**Affiliations:** 1 Department of Bionanosystem Engineering, Graduate School, Jeonbuk National University, Jeonju 561-756, Korea; 2 Division of Mechanical Design Engineering, Jeonbuk National University, Jeonju 561-756, Korea

**Keywords:** magnesium, covalent bonds, anti-corrosion, biocompatibility, bovin serum albumin

## Abstract

Herein, we describe precisely a covalent modification of pure magnesium (Mg) surface and its application to induce in vitro osteogenic differentiation. The new concept of a chemical bonding method is proposed for developing stable chemical bonds on the Mg surface through the serial assembly of bioactive additives that include ascorbic acid (AA) and bovine serum albumin (BSA). We studied both the physicochemical and electrochemical properties using scanning electron microscopy and other techniques to confirm how the covalent bonding of BSA on Mg can, after coating, significantly enhance the chemical stability of the substrate. The modified Mg-OH-AA-BSA exhibits better anti-corrosion behavior with high corrosion potential (E*_corr_* = −0.96 V) and low corrosion current density (I*_corr_* = 0.2 µA cm^−2^) as compared to the pure Mg (E*_corr_* = −1.46 V, I*_corr_* = 10.42 µA cm^−2^). The outer layer of BSA on Mg protects the fast degradation rate of Mg, which is the consequence of the strong chemicals bonds between amine groups on BSA with carboxylic groups on AA as the possible mechanism of peptide bonds. Collectively, the results suggest that the surface-modified Mg provides a strong bio-interface, and enhances the proliferation and differentiation of pre-osteoblast (MC3T3-E1) cells through a protein–lipid interaction. We therefore conclude that the technique we describe provides a cost-effective and scalable way to generate chemically stable Mg surface that inherits a biological advantage in orthopedic and dental implants in clinical applications.

## 1. Introduction

Implantable scaffolds that can inherit a remarkable biological and physiological bio-interface to host stem cells are rapidly expanding their applications towards clinical studies in bone tissue engineering [1,2]. Such scaffolds have a tendency to acquire biomimetic behavior that is akin to those of the native host tissue for absorbing proteins and biominerals, and interacting with extracellular matrix (ECM) [3]. Importantly, the biomimetic scaffolds are liable to complement wound healing systems or tissue engineering, which leads to the restoration of tissue functions, enhances stem/progenitor cells regeneration, and regulates the delivery of the cell growth factors and cytokines [4]. The principal determinants of the functional success of biomedical implants are basically their biocompatibility, natural integrity with the host tissue, and bioresorbability [5]. In bone tissue or hard tissue engineering, strong mechanical behavior, osteoinductivity, osteoconductivity, immune-compatibility, and fast-corrosion resistance with long-term stability are crucial properties at the artificial graft level [6,7]. Various materials, including metals and their alloys, natural and synthetic polymers in two-dimensional (2D) and three-dimensional (3D) structural frameworks in the form of nanofibers, and hydrogels in hybrid nanostructure confer physicochemical and biological substitutes in bone tissue repair [8,9,10,11].

Common interests in engineering biologically active and load-bearing scaffolds have been extensively explored, particularly which scaffolds can be tailored to provide a suitable structural framework for supporting cellular attachment leading to cell differentiation and tissue formation [12,13]. These properties make Mg highly attractive for the orthopedic implantable device for fracture repair with substantial capabilities to improve the regeneration and reconstruction of bone tissue by preventing implant loosening, potential infection, morbidity in the vicinity of the implantable site, and premature failure after surgery [14]. However, a rapid degradation or increase in the corrosion rate, and presumably hydrogen production from pure magnesium and its alloys in vivo do not provide prolonged mechanical support, along with an increase in alkalinity after the surgical procedure, and as a consequence, failure of bone-fracture healing [15]. Over the past several years, extensive efforts and approaches have been employed to overcome a number of inherent limitations exhibited by Mg and its alloys, including rapid corrosion, which presents a challenge for orthopedic, dental, and prosthetic applications [16,17,18]. The treatment of Mg surface to obtain Mg scaffolds with osteo-friendly biomimetic materials that include β-tricalcium phosphate (β-TCP), hydroxyapatite (HA), and chitosan, through dip-casting and immersion methods, has been studied to enhance osteogenesis and osteoimmuno-modulatory behavior [19,20]. The supplementation of these materials is known to be beneficial to subjects with osteoporosis. However, such traditional approaches are not exciting enough to be considered, as they are unable to effectively improve the anti-corrosion behavior of the surface-modified Mg and its alloy. Furthermore, various techniques, such as plasma spraying, sol–gel coating, electrophoretic deposition, sputter deposition, and spin coating, have used foreign metal oxide or particles (silica titanium, zirconium, aluminum) on magnesium surface to improve corrosion resistance behavior [21,22]. However, the use of these techniques and materials has limitations, such as low chemical stability, poor ionic conductivity, non-uniform shape and size, accumulation of nanoparticles, and rapid degradation. These are the main drawbacks that arise from the possible release of metallic nanoparticles/ions directly into cells during in vivo treatment, and which impede the cellular metabolism. Thus, they alter the cellular activity, including proliferation and differentiation, leading to the poor stimulation and upregulation of ECM, and intracellular changes such as disruption of the cellular metabolism and organelle integrity. Such changes are expected to give rise to gene alteration [23,24]. Moreover, synthetic polymers, such as polycarpolactone (PCL), poly (lactic-co-glycolic acid (PLGA), and polylactic acid (PLA), were coated on Mg surface through a layer-by-layer or multilayer system to improve its biocompatibility and anti-corrosion behavior. However, limitations still exist, including easy peeling off from the Mg surface due to only physical attachment, high cost of materials, and more hydrophobic membranes, which cause very slow diffusion of ECM and other biomolecules through the implant interfacial microstructure, resulting in the retardation of cell attachment, growth, and proliferation in vitro and in vivo [25,26].

Herein, we report the potential application of surface-modified magnesium for in vitro bioimplant in bone tissue engineering using MC3T3-E1 as model cells, instead of using Mg alloy. A simple and cost-effective chemical modification method was applied to generate an ample supply of active chemical moieties that are susceptible to forming strong covalent bonds on the Mg surface. However, fast degradation rate of pure Mg probably impedes the healing process. Thus, the surface-functionalized Mg (Mg-OH-AA-BSA) via successive treatments of ascorbic acid, followed by bovine serum albumin, was confirmed by comprehensive results of its strong anti-corrosion behavior, surface-controlled biological fixation of biomolecules or proteins, and long-term physiological stability. In addition, the bio-interface promotes osteoblastic differentiation.

## 2. Experimental Section

### 2.1. Materials

Two pure magnesium rods of (12.7 and 7.9) mm diameter (purity = 99.9%, Alfa Aesar, S.korea). Sodium hydroxide (NaOH), and L-Ascorbic acid (C6H8O6, mw = 176.12, >99.0%) were purchased from Sigma-Aldrich, S. Korea. Bovine serum albumin Fraction V (Roche Diagnostics GmbH, Mannheim, Germany) was obtained for the surface treatment of Mg. The simulated body fluid (SBF) solution was prepared in accordance with the previously reported literature [10]. Regents in aqueous solution were prepared in ultra-pure water obtained from a Millipore-Q-system. All of the chemicals and reagents were of analytical grade, and were used as received, without any further purification.

### 2.2. Sample Preparation

**Scheme 1:**
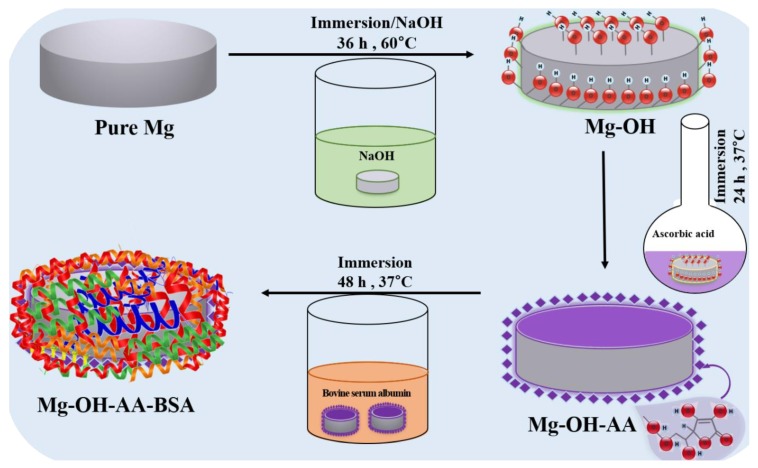
A diagrammatic representation of the modification on pure Mg surface via layer-by-layer formation of chemical bonds into Mg-OH-AA-BSA for enhancing anti-corrosion and osteo-inductive property.

The Mg rod of 12.0 mm diameter and 5.0 mm height was taken for successive surface modification, termed surface chemical treatment. Prior to the chemical treatments, the surface of each Mg specimen was polished successively with (400, 800, 1200, and 2000) grits (SiC), followed by ultrasonic bath in ethanol and water for 10 min in each step, to remove any remaining residue from the Mg surface, and dried in nitrogen atmosphere for 4 h. Scheme 1 shows the surface modification of Mg. Briefly, the pure Mg was first treated with 5.0 M NaOH in aqueous solution for 48 h at 60 °C, to generate a large number of hydroxyl (−OH) groups on the Mg surface associated with passivation. Then, it was taken out from the solution, and washed several times with DI water. The dried Mg-OH sample was treated with 0.02 M AA for 24 h at 37 °C prepared in ethanol. The treatment of AA with Mg-OH induces adequate chemical bonding through –OH and –COOH bonds, in order to form protective layers. Subsequently, the AA-treated Mg substrate was further immersed in 0.5 mM BSA for 48 h at 37 °C. The BSA forms strong bonds through –NH_2_ groups and hydrogen bonds; as a consequence, the fast corrosion rate of Mg is reduced. This behavior has made Mg-OH-AA-BSA an ideal candidate for the most biocompatible, highly protective nanoscale layer structure that enables a substantial enhancement of anti-corrosion behavior. Similar processes were applied for the surface modification of pure Mg of dimension 1.0 cm (diameter) × 0.5 cm (height), to evaluate physiological degradation after measuring hydrogen evaluation.

### 2.3. Materials Characterization 

#### 2.3.1. Physical Characterization

The surface morphologies of pure and treated Mg samples were observed by field-emission scanning electron microscopy (FE-SEM, Carl Zeiss Supra—40 VP, Oberkochen Germany). The crystallinity of the samples was characterized using X-ray diffraction (XRD, Rigaku Japan) with Cu Kα (λ = 1.542 Å) radiation over a range of Bragg angle (2θ) of (10–90°). Newly formed bonds were also confirmed by Fourier Transmission Infrared Spectroscopy (FT-IR) using Perkin Elmer Spectrum GX (Waltham, MA, USA). Atomic Force Microscopy analysis (AFM, Multimode 8, Bruker, Santa Barbara, CA, USA) was conducted to evaluate the surface roughness of the sample before and after the potentiodynamic corrosion test. Water contact angle (WCA) was measured by water contact angle meter (GBX-Digidrop, Romans sur Isere, France) at room temperature (RT) in static drop methods, where distilled water of 5 µL droplet was dropped on the surface of each sample, and the angle was measured at (1, 3, and 5) s.

#### 2.3.2. Electrochemical Measurement

Electrochemical analysis, such as the determination of the open circuit potential (OCP) vs. time, potentiodynamic polarization test, and electrochemical impedance spectroscopy, were conducted using ZIVE SP1 Potentiostat/Galvanostat/EIS electrochemical analysis (WonATech Co. Ltd. Seoul, Korea) to determine the OCP of the cell running for 30 min, the corrosion resistance behavior of the prepared magnesium samples, and the charge transfer property of the samples, respectively. We used a three-electrode electrochemical configuration, where Mg or modified Mg samples were used as working electrodes, saturated calomel electrode (SCE) was used as reference electrode, and platinum mesh was used as counter electrode. Inorganic salts of magnesium sulfate, calcium chloride, and sodium bicarbonate, along with Hank’s balanced salts (H2387, Sigma Aldrich, Korea), were dissolved in 1 L of DI water (pH 7.4) in accordance with the literature [27], and the solution-simulated body fluid (SBF) was used as electrolyte for each electrochemical measurement at RT. The potentiostatic electrochemical impedance spectroscopy (EIS) was recorded from all surface-modified and pure Mg in SBF solution (pH 7.4) at an amplitude of 10 mV and zero bias potential in the frequency range (100 kHz–1 mHz). All samples were stabilized in SBF for 1 h 30 min at 37 °C, prior to electrochemical measurement. The SBF used as electrolyte was also purged with pure nitrogen (N_2_) for 15 min, to reduce the oxygen that dissolved on the electrolyte before each measurement. The corrosion potential was measured via Tafel extrapolation polarization from potential scanning from (−2 to 0) V vs. OCP at a constant sweep rate of 1 mV/s. All the samples with effective interface surface area of 0.875 cm^2^ were examined for their electrochemical properties.

#### 2.3.3. Hydrogen Evolution Test

The corrosion behavior of the as-prepared samples was evaluated through the conventional immersion test method. The volume of hydrogen (H_2_) evolved at different time points was measured, and the degradation rate (DR) was calculated at normal temperature and pressure (NTP) using the relation, $\left(\mathrm{DR}\right)\;=\frac{PV}{RT}\left(\frac{M}{At}\right),$ where P, V, R, T, M, A, and t correspond to the normal pressure at RT, volume of H_2_ evolved in mL, universal gas constant, RT in Kelvin, molar mass of Mg, active surface area, and exposure time, respectively [28]. The process was carried out at different time points to monitor the degradation of magnesium. Each tested sample (n = 3) was separately immersed into an air-tight system using an eudiometer containing 500 mL SBF, and then incubated at 37 °C for a total of 8 days. The volume of H_2_ from each sample was measured at 6 h and then at 12 h intervals i.e., at (6, 12, 24, 36, and up to 192 h). The corrosion rate was calculated on the last 24 h of day 8. Pure Mg with dimension 1.0 cm (diameter) and 0.5 cm (height) was taken as reference sample.

#### 2.3.4. In Vitro Cells Interaction

Pre-osteoblast (MC3T3-E1) cell lines derived from mouse Clone 4, ATCC CRL-5293 were first cultured in Minimum Essential Medium (α-MEM, Hyclone^™^, Thermo Fisher Scientific, Logan, UT, USA) under incubation at 37 °C and 5% carbon dioxide (CO_2_), in accordance with the previously reported literature [29,30]. Briefly, cells at almost 85% confluency were harvested, then seeded (under UV light for overnight) onto the center of sterile tissue culture plate (TCP) and Mg-OH-AA-BSA scaffold at a density of 3 × 10^4^ cells/well. The cells were cultured for different time points (for example, (1, 3, and 7) days). During this time, the cell medium was replaced every second day. Furthermore, a cell viability test was conducted to evaluate the cells’ proliferation, survivability, and cytocompatibility on the as-prepared scaffolds, and were continually monitored up to 7-day period using the cell counting kit-8 (CCK-8) at intervals of (1, 3, and 7) days of cell culture. At preferred time points, the CCK-8 test solution was added to each 24-well plate, and the cells incubated for 2 h at 37 °C incubator with 5% CO_2_. The measurement of the absorbance was checked at 450 nm using a microplate reader (Tecan, Austria) in 96-well plate. Furthermore, we also confirmed the cell growth and viability using a colorimetric, 3-(4,5-dimethylthiazol-2-yl) 2,5 diphenyl tetrazolium (MTT, Dojindo Molecular technologies) assay at interval of 1, 3 and 6-days of cell culture (Supplementary Figure S1).

#### 2.3.5. Cell Morphology Observation

The morphology of the MC3T3-El cells grown in vitro was evaluated by fixing the cells with 4% glutaraldehyde solution for 1 h on days 1, 3, and 7 post-seeding, followed by washing with PBS to remove the non-adherent cells. Again, cells were washed with PBS, followed by dehydration in a graded series of ethanol concentrations of (20%, 30%, 50%, 70%, and 100%) for 10 min each. The cell samples were dried for 12 h on clean bench, before observing cell morphology by cell scanning microscopy at different magnification (JSM-6400F, Tokyo, Japan). In addition, to obtain the confocal images, the cells cultured on the Mg-OH-AA-BSA scaffolds were washed with PBS, after aspiration of media from 24-well plate. The cells were fixed with 4% paraformaldehyde for 10 min at RT, and permeabilized in 0.2% triton (X-100) for 2 min. The cells were blocked with 1% human serum albumin for 30 min. The scaffolds attached with cell were consecutively stained by Rhodamine-phalloidin for cytoplasm (20 min), and DAPI for cell nuclei (5 min), at RT in dark condition, followed by washing with PBS. The fluorescence images of the cells were taken by confocal microscopy (Zeiss LSM 800 Airyscan, Carl Zeiss Micro Imaging Inc., Thornwood, NY, USA).

#### 2.3.6. ALP Activity

ALP analysis was conducted according to the reported literature [31]. Briefly, the cells at a density of 2 × 10^4^ cells/well on 24-well plates were cultured on TCP (as control) and Mg-OH-AA-BSA scaffold (n = 3) at different time periods of (6 and 10) days, separately. Cells were lysed with 1% Triton X-100 solution for 15 min. The supernatants collected after centrifugation were transferred into 96-well-plate, and used to examine the ALP activity after treatment with p-nitrophenyl phosphate (pNPP). The colorimetry measurement at 450 nm via microplate reader was recorded. The total protein content was evaluated using a BCA protein assay, and ALP levels were normalized to the total protein content.

#### 2.3.7. Type I Collagen Analysis

The quantity of collagen type I protein secreted from MC3T3-E1 cells seeded on TCP (as control) and Mg-OH-AA-BSA scaffold (n = 3) at (7 and 14) day time intervals was evaluated using Sirius Red/Fast Green Collagen Staining Kit (9046, Chondrex Inc. Redmond, WA, USA), on the basis of company protocol. Cells were cultured according to the aforementioned method, and washed with PBS solution, followed by Kahle fixation (a mixture of DI water, 95% ethanol, formaldehyde, and acetic acid) for ten minutes at RT. The fixed cells were treated in dye solution for 30 min, and washed repeatedly. The color of the stain was eluted by dye extraction buffer. The absorption of eluted dye solution was quantified under spectrophotometry at (540 and 605) nm. The type I collagen secretion was calculated according to the manufacturer’s protocol.

#### 2.3.8. Statistical Analysis 

Data were evaluated using one-way analysis of variance (one-way ANOVA) followed by post hoc Tukey comparison test at a 95% confidence level, and presented as mean ± standard deviation (SD, n = 3). Statistical significance was considered when * *p* < 0.05, and *** *p* < 0.001. 

## 3. Results and Discussion

### 3.1. Surface Analysis

**Figure 1:**
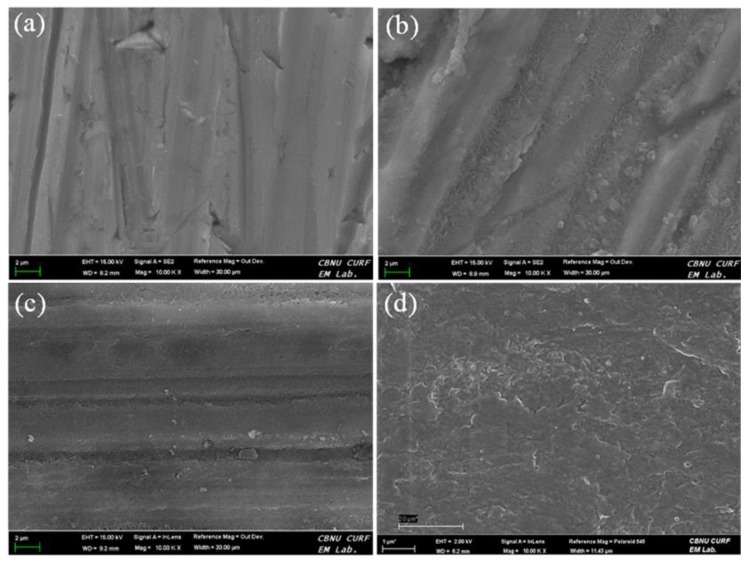
FE-SEM images showing surface topography of (**a**) pure Mg, (**b**) Mg-OH, (**c**) Mg-OH-AA, and (**d**) Mg-OH-AA-BSA before the evaluation of potentiodynamic polarization test.

Figure 1 shows the FE-SEM images through which the surface topography of magnesium substrates with multilayer coating of chemical moieties including ascorbic acid and BSA were studied, before immersion in SBF solution. A smooth and shining surface of pure Mg was observed (Figure 1a), due to its high crystallinity. Figure 1c,d show the surface of Mg samples that were coated or treated successively through chemicals by physical or chemical adsorption technique in accordance with the formation of strong chemical bonding with NaOH, AA, and BSA. After Mg-OH-AA was treated with BSA, the roughness of surface slightly decreased. The nanoscale of the BSA layer on the outer surface made the surface smoother, as compared to Mg-OH and Mg-OH-AA surface, confirming that the hydrophobic protein moieties were strongly attached to the Mg-modified surface.

**Figure 2:**
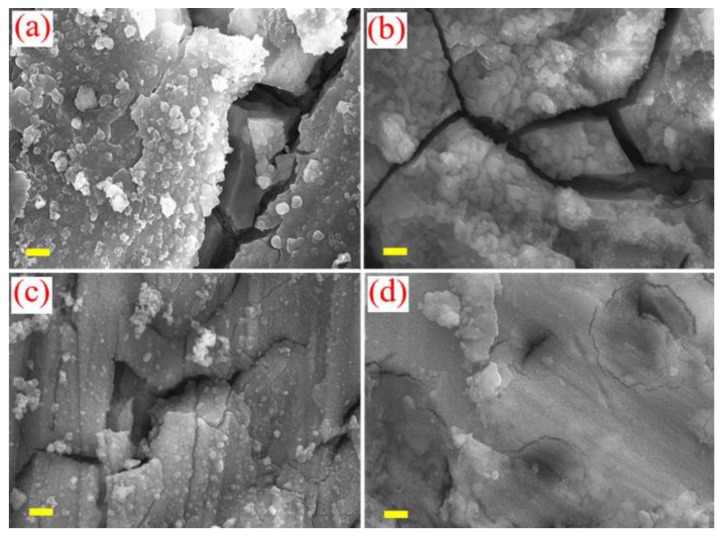
Representative FE-SEM surface morphology of (**a**) the pure Mg, (**b**) Mg-OH, (**c**) Mg-OH-AA, and (**d**) Mg-OH-AA-BSA, after potentiodynamic polarization test conducted in SBF solution at 37 °C. (Scale bar = 1 µm).

Figure 2 shows the surface corrosion morphologies of Mg and Mg modified samples after potentiodynamic corrosion test. The samples were immersed in SBF for 2 h prior to the Potentiodynamic test. The pure Mg showed microcracks and large surface roughness due to its high reactivity, but the passivation of Mg increased after treating with NaOH by oxide layers. The electrovalent bonding between Mg and hydroxyl groups decreases the reactivity in the aqueous phase, and relatively lower cracks were observed (Figure 2b). Moreover, the Mg-OH surface covered with AA groups further reduced the microcracks (Figure 2c). It is obvious that the formation of several chemical bonds, including covalent and hydrogen bonds between Mg-OH and AA, led the Mg-OH-AA to show less sensitivity towards pitting corrosion.

Importantly, our interest is more concerned with improving the anti-corrosion property of Mg after surface modification. Figure 2d shows that the Mg-OH-AA-BSA sample revealed significant improvement in surface smoothness, as compared to the other samples. The surface morphology of Mg-OH-AA-BSA was remarkably protected from degradation, and showed very many less cracks, suggesting chemical inertness as a consequence of the strong −OH, −COOH, −NH_2_, and hydrogen bonds between BSA and AA. 

**Figure 3:**
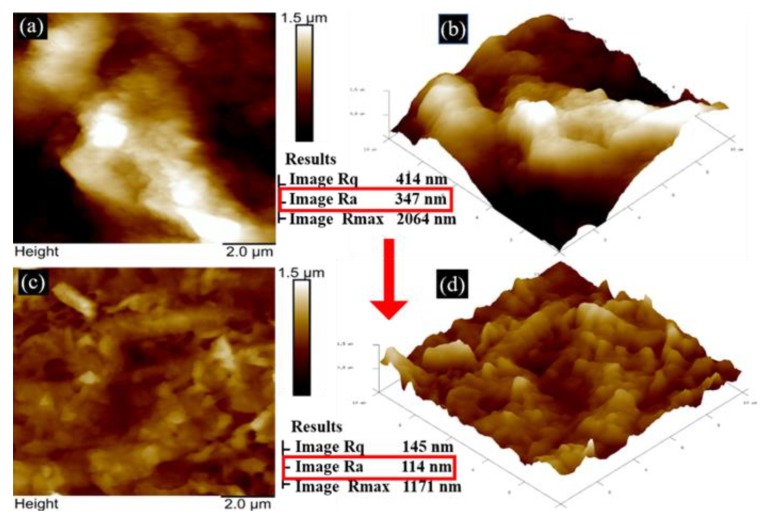
Atomic force microscopy images in 2D and 3D forms of (**a**) and (**b**) pure Mg, and (**c**) and (**d**) Mg-OH-AA-BSA, after polarization test in SBF at 37 °C.

AF microscopy results in Figure 3 confirm that the average surface roughness (Ra ~114 nm) value and Rq (~145 nm) value in the two-dimensional (2D) and three-dimensional (3D) micro/nano structure of Mg-OH-AA-BSA is almost three-fold less than that of the pure Mg, which indicates that after the strong encapsulation by BSA, the BSA-modified Mg has uniform and smooth surface. This result suggests that the appropriate roughness on Mg-OH-AA-BSA sample surface exhibits preferentially for stem cell attachment and the proper cellular function, and also provides a suitable interface for the adsorption of protein/lipid or extracellular matrix (ECM).

**Figure 4:**
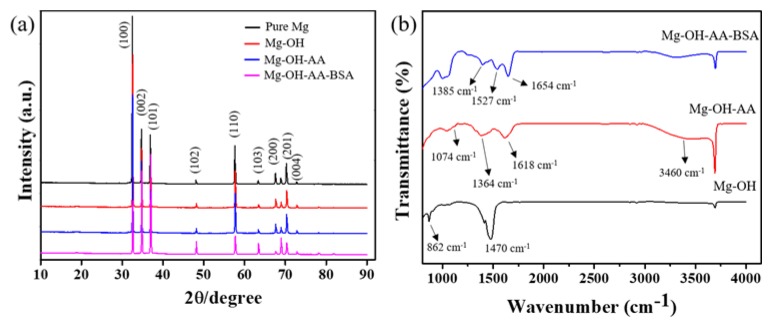
(**a**) XRD analysis of pure Mg and other surface modified Mg samples. (**b**) FT-IR spectra of as-prepared samples surface.

Figure 4a shows the XRD patterns of the as-prepared materials. The hexagonal phase crystallography of pure Mg was confirmed by the diffraction peaks profiles in several lattice planes (100, 002, 101, 102, 110, and 200), corresponding to 2θ = (32.61, 34.89, 37.19, 48.25, 57.87, and 67.62°), respectively. These values are consistent with the values reported previously in the literature [32]. These values in degrees associated with planes indicate the purity of Mg without the presence of oxide and hydroxide layers on the Mg surface. However, while no notable peaks were observed on Mg (OH)_2_, the multiple diffraction peaks that appeared from Mg (OH)_2_ were also in agreement with the hexagonal structure of Mg (OH)_2_ (JCPDS 7-239). The successive surface modification of Mg by AA followed by BSA had no significant effect on the crystallography of Mg. Interestingly, the peak intensities decrease in the modified Mg in respect of the presence of nanoscale layers of BSA that are strongly coated on the Mg-modified surface.

Furthermore, the FT-IR spectroscopy analysis depicted in Figure 4b describes the adsorption peaks at different frequency region in the range of 800 to 3700 cm^−1^. The absorption peak at ~862 cm^−1^ is assigned to Mg-O bonds in vibrational mode. A notable peak around 1470 cm^−1^ may indicate the presence of Magnesium hydride (Mg-H) bonds [33]. It might have been possible that after the treatment of Mg in strong alkaline solution (sodium hydroxide) at high temperature (~80 °C), the Mg ^++^ ions at the surface of Mg could combine with hydride (H^−^) ions (produced from the oxidation of sodium hydroxide). This could develop as the formation of a new Mg-H bond. In addition, the adsorption bands at 1053 and 3700 cm^−1^ correspond to the formation of Mg-O and Mg-OH, respectively [34]. However, well-defined bands that appear at 1074, 1364, and 1618 cm^−1^ confirm the C-O-C, enol-hydroxyl groups, and C=C, respectively from the ascorbic acid on the Mg surface [35]. It is worth noting that a wide band intensity at around 3,359 cm^−1^ indicates the presence of −OH in AA that bonded to Mg–OH through the ample hydrogen bonds. We further confirmed that the conjugation of BSA to AA modified Mg through the strong adsorption band of BSA on the Mg surface. The characteristic bands of BSA at 1385, 1527, and 1654 cm^−1^ are attributed to C-N stretching, N-H bending mode of amide II, and C=O stretching mode of amide I, respectively [36]. These particular band intensities present in Mg-OH-AA-BSA substrate indicate the nanoscale layer of BSA uniformly encapsulated on the Mg surface through strong −NH_2_, C=O, and covalent bonds.

**Figure 5:**
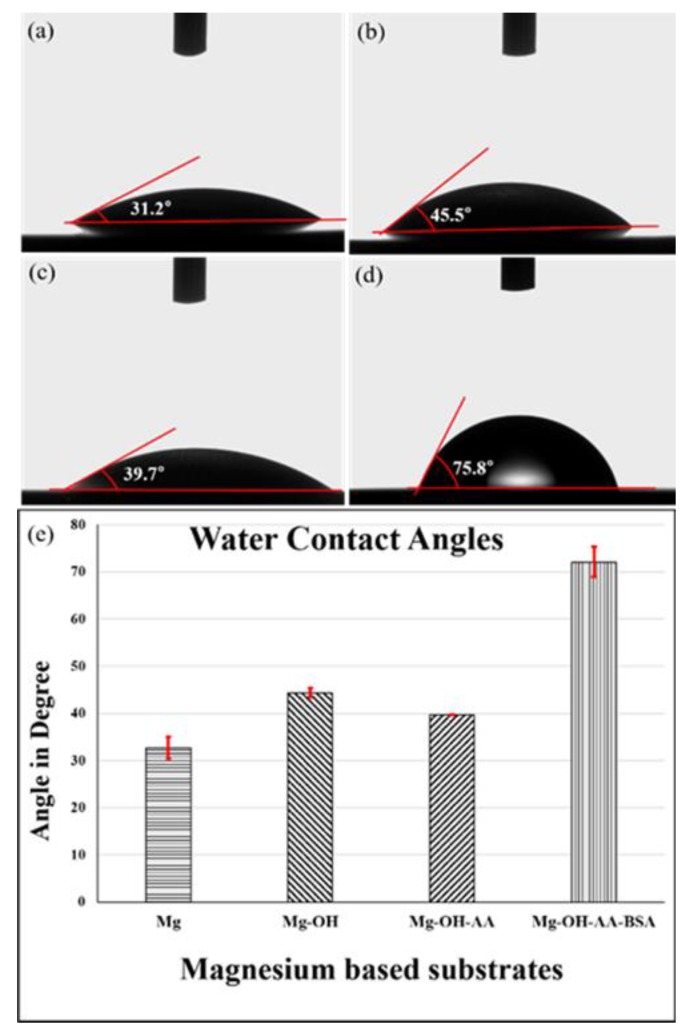
Water contact angle values of the samples including (**a**) pure Mg, (**b**) Mg-OH, (**c**) Mg-OH-AA, and (**d**) Mg-OH-AA-BSA at RT. (**e**) histogram showing the average contact angle value of each substrate (n = 3). The WAC value of each sample was obtained before potentiodynamic corrosion measurements.

To investigate the surface-interface of the as-prepared Mg and Mg surface modified samples with water molecule, we performed static water contact angle measurement, as shown in Figure 5. Pure Mg exhibited a lower WCA value (~32.2°) compared to the WCA values of Mg-OH (~45.5°) and Mg-OH-AA (~39.7°). It is most important that the ample oxide layers on the reactive Mg at RT are susceptible to forming H-bonding with H_2_O. However, the −OH and oxide moieties on Mg–OH, layer passivate the modified sample more than the pure Mg for the formation of H-bonding. Consequently, increases in water contact angle were observed on Mg–OH. Notably, the large number of water loving groups (−OH and C=O) in ascorbic acid at Mg layer lower the WCA of Mg-OH-AA (Figure 5c). In addition, the effective WCA was examined on surfactant-bonded BSA (protein) on Mg surface. We know that BSA, of amphiphilic nature, absorbs H_2_O forcefully, and reduces the interfacial tension by immobilization in the water. Thus, the hydrophobic interaction increases on BSA-modified Mg (Figure 5d) is confirmed by the value of WCA on Mg-OH-AA-BSA (~75.8°). The contact angles value of each substrate is also expressed on the histogram (Figure 4e), indicating the layer of chemical bonding of BSA on the modified Mg surface.

It is obvious that the hydrophobic proteins have great stability, and biologically function as nonpolar interaction with ECM, which helps to bind other lipids and proteins [37]. Such materials are more advantageous for cytocompatibility, and could be preciously applied for the development of bioimplants in hard tissue engineering (for example, dental or bone graft) as artificial scaffolding.

**Figure 6:**
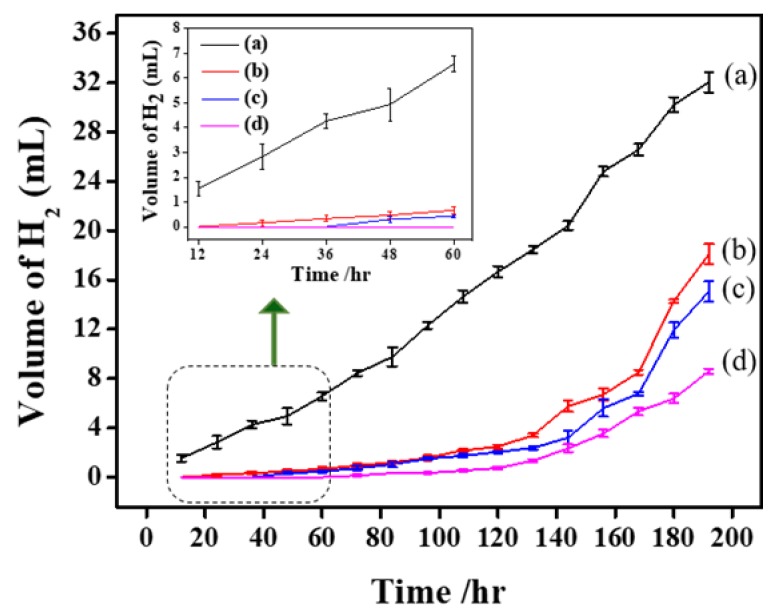
(**a**) Hydrogen evolution from (**a**) pure Mg, (**b**) Mg-OH, (**c**) Mg-OH-AA, (**d**) Mg-OH-AA-BSA substrate (n = 3). Inset shows the volume of H_2_ gas evolved within 60 h.

Figure 6 expresses the volume of hydrogen evolved from the Mg samples that was measured. The curve (d) depicts that no H_2_ was evolved until 60 h from the Mg-OH-AA-BSA sample. However, at the end of day 3, the degradation rate on the basis of H_2_ evolution was calculated, and found to be 2.7 mg cm^−2^ day^−1^ in pure Mg. In contrast, the surface-modified Mg samples showed gradually lower degradation rates, and basically, the Mg-OH-AA-BSA has the significantly lowest corrosion rate (of 0.04 mg/cm^2^.day), indicating that the nanolayer of BSA has strongly protected the Mg surface through effective chemical bonding to inter-molecular chemical moieties. At day 8, the BSA-modified showed little increase in corrosion rate (2.7 mg cm^−2^ day^−1^), as compared to day 3. The increase in corrosion rate might have been the consequence of small pit formed on Mg, so that the release of metal ions increased from the mechanism of the chemical degradation reaction given by: Mg + H_2_O → Mg^2+^ OH^−^ +H_2_↑. The pure Mg released more Mg ions in SBF solution, but was suppressed on Mg-OH-AA-BSA, which is only caused by the compact layer of BSA exhibiting physical and chemical stability [38].

### 3.2. Electrochemical Study

**Figure 7:**
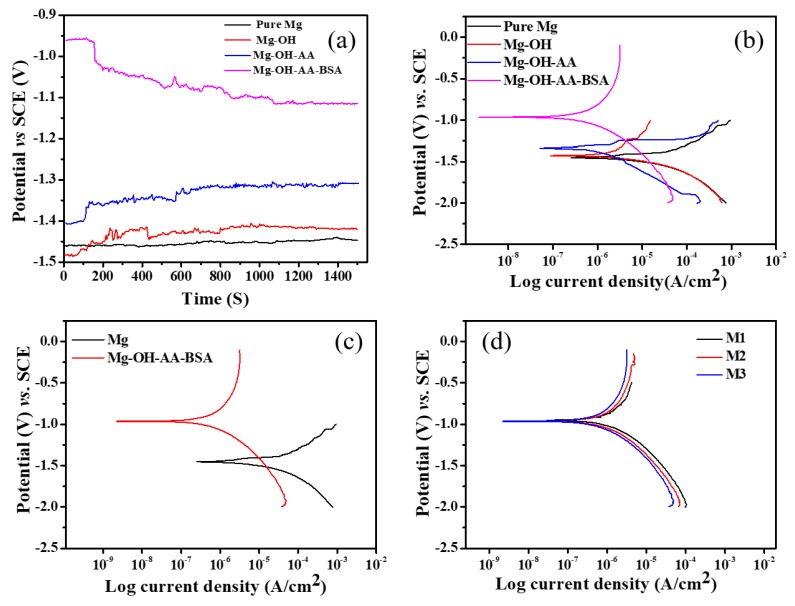
Electrochemical corrosion study. (**a**) OCP vs. time; (**b**) potentiodynamic polarization curves; (**c**) potentiodynamic polarization curves of Mg and Mg-OH-AA-BSA only; and (**d**) polarization curves obtained from Mg-OH-AA-BSA (n = 3). All samples were treated in SBF solution at 37 °C for 1 h 30 min, before analytical measurements.

First, to evaluate how the chemical moieties linked with BSA influence the anti-corrosion performance of Mg samples, we choose all surface-modified Mg, including pure Mg for OPC measurement in Figure 7a. Chemically, the outer surface of the modified Mg-OH-AA-BSA is composed of multiple functional (−OH, −NH_2_, and −COOH) groups associated with ample of inner- and inter-ionic bonding and other bonds (for example, hydrogen bonding, and amide–hydrogen bonding). These interconnected molecules form a layered-like structure on the Mg surface. The samples with a different layered structure showed variable OCP of the cell. The OCP (vs. time) results from the different samples illustrate that the Mg has lower cathodic potential, while the anodic potential gradually increased, and Mg-OH-AA-BSA has stable and the largest anodic potential. The decrease in cathodic potential by Mg (~−1.45 V) is assigned to the rapid dissolution of bare Mg. It is worth mentioning here that the higher anodic potential exhibited by Mg-OH-AA-BSA at around ~−1.1 V is the lowest OCP value of the running cell so far reported in the literature [39,40]. The uniform coating of BSA has the potential advantage of suppressing the corrosion reaction, and reducing the H_2_ evolution rate. This result indicates that under such chemical modifications, the anti-corrosion behavior of Mg-OH-AA-BSA showed much better performance than that of other commonly employed methods [41]. 

Figure 7b shows the potentiodynamic polarization curves obtained from the different samples. The Mg-OH-AA-BSA showed the highest corrosion potential (*E_corr_*) value, along with the lowest corrosion current density (I*_corr_*). Table 1 lists the electrochemical corrosion parameters determined from the Tafel slope extrapolation. The free corrosion potential of the surface-modified Mg including pure Mg showed the order Mg < Mg-OH < Mg-OH-AA < Mg-OH-AA-BSA. In contrast, the I*_corr_* values from the samples were in reverse order. This indicates that the formation of chemical bonds after consecutive modification of Mg surface enhanced the electrochemical stability in Mg-OH-AA-BSA. Meanwhile, the more negative value of I*_corr_* in BSA-modified Mg showed the best anti-corrosion property, which is attributed to the strong protective layer of BSA. In general, proteins interact with metals through adsorption and chelation, and improve the anti-corrosion behavior. However, BSA has no ability to protect the anti-corrosion behavior of first row d-block metals, due to its ability to form complexes, and dissolution of metals can occur by protein-catalyzed reaction [42], but formation of oxide and hydrate layers in Mg surface enhance the bond strength with BSA. Consequently, both the cathodic reaction of hydrogen evolution and the dissolution of Mg^2+^ (anodic oxidation) can be significantly reduced. In addition, the Mg-OH-AA-BSA showed almost no pitting potential, as compared to other modified samples, including pure Mg. The result obviously confirms that the chemical modification of Mg surface through the formation of covalent bonds, amine bonds, and hydrogen bonds has a true and stable modification technique, and was proven by the remarkably higher cathodic potential (−0.96 V) value of Mg-OH-AA-BSA. Moreover, the inhibition efficiency η (%) of the BSA-modified Mg was calculated and found to be 98.08%, indicating the suppression of cathodic electrochemical corrosion behavior, which is also in agreement with the FESEM images (Figure 2d). Moreover, the E*_corr_* and I*_corr_* observed on Mg-OH-AA-BSA (n = 3) showed almost 97.23% reproducibility, indicating the good reproducibility of the prepared samples (Figure 7d).

**Figure 8:**
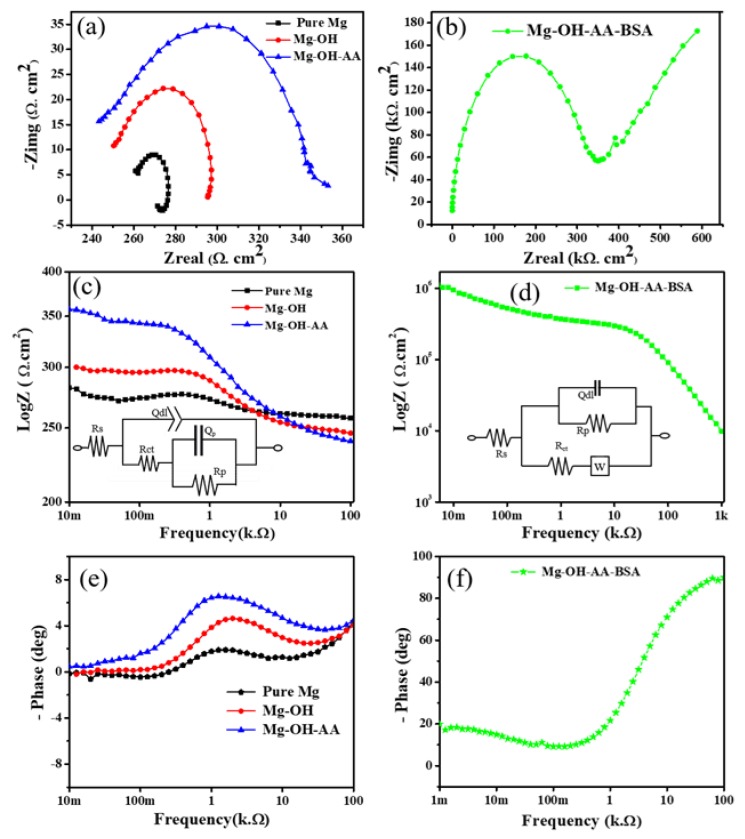
Electrochemical impedance spectroscopy analysis. (**a**,**b**) Nyquist plots, and (**c**–**f**) Bode plots of the samples measured in SBF solution at 37 °C (insets represent the equivalent electrical circuit of EIS spectra obtained from (**a**) and (**b**), respectively).

Figure 8 depicts the Nyquist and Bode plots that were made in order to evaluate the electrode/electrolyte interface in potentiodynamic polarization reaction that occurs at the electrode surface in SBF solution. The Nyquist plots illustrate that the chemical modification of Mg-OH and Mg-OH-AA showed larger impedance than that for the pure Mg. This result clarifies the formation of protective layers of oxide and hydration in association with carboxylate groups can passivate the coating layers. Importantly, the chelating behavior of BSA resists percolation of the electrolytes during cathodic polarization reaction, and significantly increases the impedance behavior. The excessive large capacitive loop (Figure 8b) exhibited by Mg-OH-AA-BSA substrate corresponds to charge transfer resistance (R*_ct_*) at higher frequency region, which indicates the best agreement with the theoretical hypothesis, suggesting a better anti-corrosion chemical modification, compared to the only oxide and hydrate layer coating layers on other samples. The chemically modified Mg-OH and Mg-OH-AA (Figure 8c,d) show a higher impedance modulus value than the bare Mg, but the value appears remarkably higher on Mg-OH-AA-BSA due to the organic barrier of proteins.

The corrosion processes were further elaborated through the equivalent electrical circuits (ECs) in the insets of Figure 6 to determine the impedance parameters in circuits. The ECs shown in Figure 8c inset represent the three samples (Pure Mg, Mg-OH, and Mg-OH-AA) as in the model: Rs-Qdl|R*_ct_*–Qp|Rp, and for Mg-OH-AA-BSA was proposed as Rs-(Qdl|Rp)|R*_ct_–*w, where Rs represents the resistance from electrolytes, and Qdl and R*_et_* the double layer capacitance and charge transfer resistance, respectively. Similarly, Q_p_ is the capacitance due to the natural oxide in the surface, R_p_ relates to the resistance attributed to the charge transfer reaction, and w represents the Warburg impedance [42]. Moreover, the phase angle diagram of Mg-OH-AA-BSA in Figure 8f approaches –89.23° at medium range frequency, which is significantly lower, as compared to other samples. These results verify the passivation of the outermost layer of materials causing the synergistic effect of oxide film covalently bonded to proteins forming a protective layer [43].

### 3.3. In vitro Cell Viability

**Figure 9:**
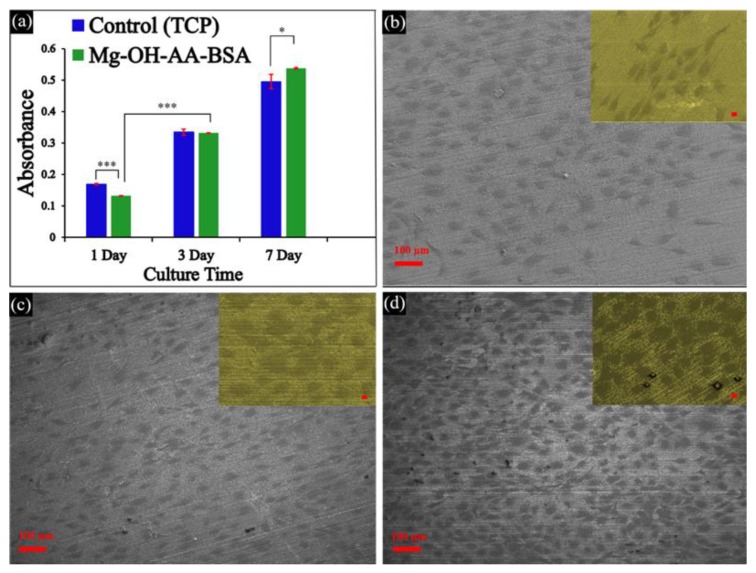
(**a**) Pre-osteoblast cell viability (cell counting Kit-8, CCK8 test) on the Mg-OH-AA–BSA (samples) and TCP (as control). (**b–d**), Representative SEM images of MC3T3-E1 cells cultured on Mg-OH-AA-BSA scaffolds surface after (1, 3, and 5) days of culture. Data presented as mean ± SD, and analyzed using ANOVA, * *p* < 0.05, and *** *p* < 0.001 (Scale bar =20 µm (insets)).

Pure Mg sample is inappropriate for long-time direct integration with stem cell during in vitro cell viability test, due to its rapid corrosion rate [7]. Thus, it is not suitable to consider for biomedical implant. In this work, a comparative analysis was conducted to evaluate the osteogenic activity of Mg-OH-AA-BSA sample in MC3T3-E1 cells through CCK-8 test result (Figure 9a). The development and proliferation of the cells on the as-prepared samples were observed at different time points. The cell viability on the samples did not show any significant difference as compared to cell grown on tissue culture plate (TCP, taken as control group). The formation of oxide layers on the Mg surface associated with chemical bonding of other multi-functional groups (NH_2_, COOH, −OH) via the assembly of chemical moieties in the serial modification method leads to the easy introduction of a strong nanoscale layer of proteins (BSA). This could promote osteogenic properties existing in the natural environment on Mg-OH-AA-BSA. Thus, the protein-based scaffolds have osteoinductive ability, which is essential for tissue regeneration and differentiation. The cells were significantly less proliferated on Mg-OH-AA-BSA than TCP on day 1. In the first 24 h, the cells were unable to adapt sufficiently to the protein-rich scaffold, due to the changing functional moieties on the scaffolds—cell interface interaction. However, the anabolic activities gradually increased on the scaffolds, and the scaffold promoted the growth and proliferation of MC3T3-E1 cells on both days 3 and 7, as compared to TCP. The outer layer of BSA on the scaffolds provides nutrients/ECM that are easily bonded to protein. The regular intake of proteins stimulates the bone cells formation, along with differentiation, and assists in suppressing bone breakdown [44]. It is worth mentioning that the Mg-OH-AA-BSA scaffold with sufficient protein provides a natural environment, and has great impact on cell functions. This was confirmed from the comparative study of the cell proliferation rate that occurred on the scaffolds and the TCP. In addition, the significant improvement (*** *p* > 0.001) of cell growth and proliferation observed on the scaffolds on days 3 and 7 supports the evidence of the strong protective layer and anti-corrosion behavior of BSA-modified scaffolds. The cells were remarkably proliferated on the scaffolds (* *p* < 0.05), compared with on the TCP, indicating that the as-prepared Mg-OH-AA-BSA scaffold demonstrated potential effects for in vitro cell adhesion, growth, and proliferation. These results attest to the relevance of the scaffolds for long-term bioimplant in association with the perfect remodeling of bone cells.

**Figure 10:**
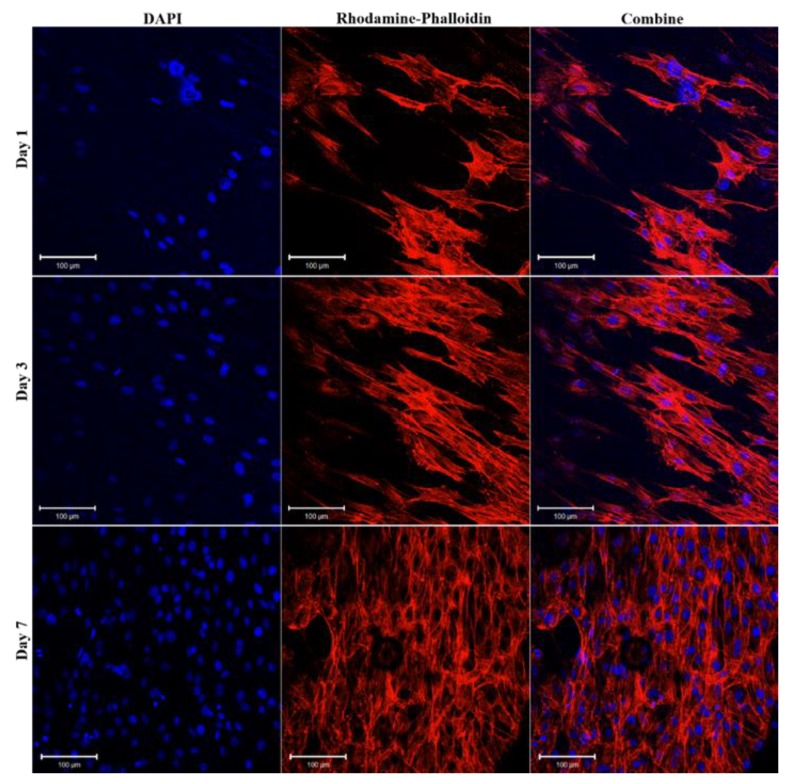
Fluorescent images of MC3T3-E1 pre-osteoblast cells attached on Mg-OH-AA-BSA scaffolds. The cells were cultured on the samples for (1, 3, and 7) days, separately. Nuclei and cytoplasm were stained by DAPI (blue) and Rhodamine–Phalloidin (red), respectively. (Scale bar = 100 µm).

Figure 9b–d and Figure 10 show the cell scanning microscopy images and confocal fluorescence images of preosteoblast cells after culturing on the Mg-OH-AA-BSA scaffold at three different time points, respectively. The cell attachment, growth, and proliferation gradually increased with prolonged period of time up to 7 days in this study. The cells were uniformly distributed with high confluency rate at 7 days, indicating the uniform coating of BSA on the surface-modified Mg enhanced the development of cells. The cellular morphology on the scaffold at day 3 clearly shows that the cells are spreading naturally and are more strongly attached on the scaffold than at day 1 (Figure 9b,c). Furthermore, Figure 9d shows the high cell density with good cell proliferation, which indicates that the scaffold exhibited better biocompatibility and strong anti-corrosion behavior.

The adhesion, distribution and proliferation of the cells represented by the fluorescence images (Figure 10) are also in agreement with the SEM images (Figure 9c,d). Here, it is clear that the chemically modified Mg surface with a protein-rich protective layer provides sufficient nutrients for the cells, and the cell functions (for example, growth and differentiation) were remarkably enhanced. Such results are the clue for the application of Mg-OH-AA-BSA bio implant bone formation.

### 3.4. Analysis of ALP Activity

**Figure 11:**
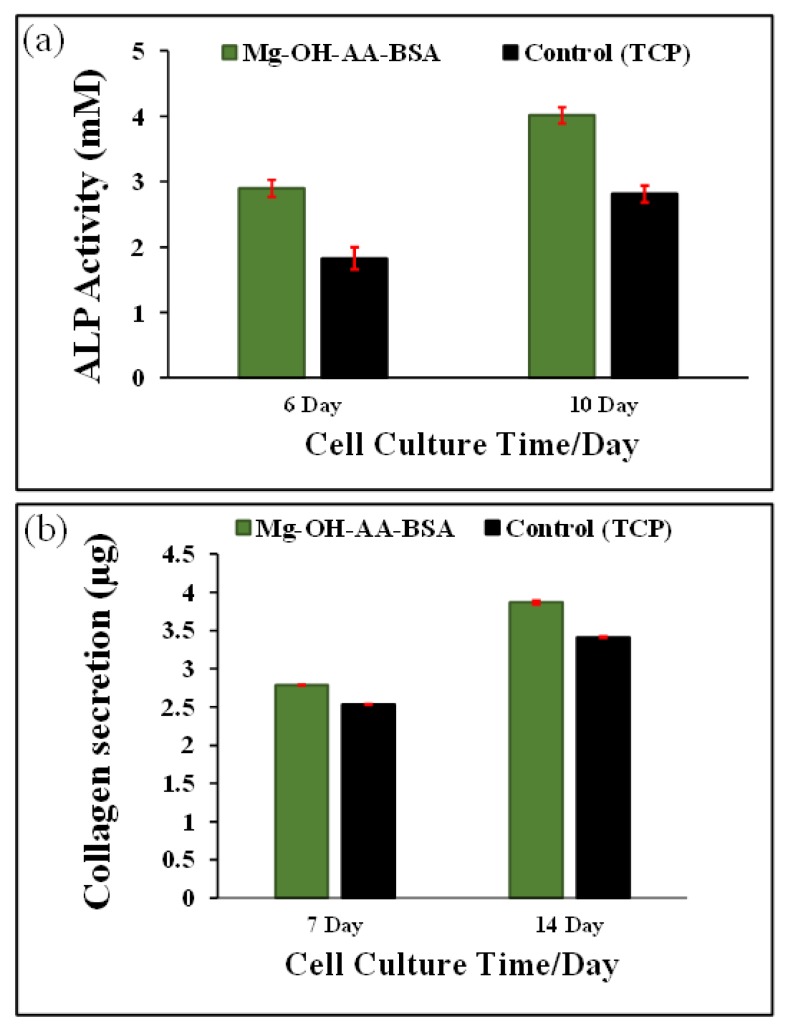
(**a**) and (**b**) ALP activity of MC3T3-E1 cells and expression of Type I collagen at various time periods, respectively.

The activity of ALP is considered to specify the presence of osteoblast cells and formation of new bone. Figure 11a shows the quantitative analysis of the ALP activity of the cells cultured on TCP and Mg-OH-AA-BSA measured by colorimetric analysis using pNPP after (7 and 14) days. The ALP activity of the cell cultured on Mg-OH-AA-BSA was found to be significantly higher than on TCP. This finding suggests that Mg-based substrate modified with BSA provides the microenvironment of bone cell and accelerates the growth of osteoblast cells, which refers to osteogenesis. The BSA provides a good combination of cell-to-bio-interface, and provides strong biocompatibility [45]. The increased ALP activity of cells cultured on Mg-OH-AA-BSA is the aftermath of the chemical modification of Mg surface via serial modification by organic moieties, which provide a controlled corrosion rate. Notably, the presence of serum-derived factors stimulates the expression of ALP to ensure osteoblastic or stem cell differentiation, and also upregulates the enzyme activity [46,47].

To further investigate the evidence of pre-osteoblast differentiation, Figure 11b shows the expression of type I collagen secretion from the cells cultured on TCP and Mg-OH-AA-BSA substrate. The bio-friendly environment of BSA on the Mg-based substrate has the ability to easily communicate through the cell-to-bio-interface, and provides a platform for the cellular activity, including growth and differentiation. As a consequence, the mature osteoblast cells secrete collagen matrix as bone ECM. The secretion of the matrix is higher from the cell’s growth on the Mg-based substrate than the TCP, indicating that the osteogenic mechanism favors the substrate.

## 4. Conclusions

In summary, we controlled the fast biodegradable property of Mg via covalent functionalization on its surface, along with the formation of nanoscale layer of BSA. The electrochemical behavior of the Mg-OH-AA-BSA substrate reveals that the formation of covalent bonds through the successive addition of functional moieties on the Mg surface prevents a fast corrosion rate of Mg. The modified Mg-OH-AA-BSA substrate showed surface smoothness and strong anti-corrosion behavior, indicating the perfect coating of BSA. The surface WCA of Mg-OH-AA-BSA sample exhibited a hydrophobic character, which is beneficial for protein–protein or protein–lipid interactions. In addition, the uniform coating of protein on the surface creates a natural environment for the development and proliferation of MC3T3-E1 cells. The Mg-OH-AA-BSA showed good osteo-compatibility and osteo-integrative performance for MC3T3-E1 cells. This novel facile idea could apply to the modification of lightweight metal, and has potential efficacy for the fabrication of bio implant devices for bone tissue applications.

## Figures and Tables

**Table 1:** The value of electrochemical parameters obtained from various samples using potentiodynamic polarization technique (calculated from Tafel plots and Nyquist plot), measured in SBF solution at 37 °C.

Samples	Polarization Parameters			Nyquist Plots				
	Ecorr	I*_corr_*	−β_c_	Rs	Rct	Rp	Qdl	Qp
	*Vs.(SCE)*	(μA.cm^2^)	(mV/dec)	(kΩ. cm^2^)	(K Ω. cm^2^)	(K Ω. cm^2^)	(µF.cm^−2^)	(µF.cm^−2^)
Pure Mg	−1.46	10.42	0.108	0.26	0.012	0.014	62.43	8
Mg-OH	−1.43	1.04	0.148	0.24	0.051	0.049	3.41	5.26
Mg-OH-AA	−1.34	0.61	0.135	0.23	0.11	0.068	1.62	2.1
Mg-OH-AA-BSA	−0.96	0.20	0.274	11.66	344.52	283.51	−	−

‡ 1.951 × 10^−7^ (Ω) Warburg constant of Mg-OH-AA-BSA sample.

## Supplementary Materials

The following are available online at https://www.mdpi.com/2073-4360/12/2/439/s1, Figure S1. Cell growth and viability analysis using MTT assay to confirm the proliferation of pre-osteoblast (MC3T3-E1) cells on Mg–OH–AA–BSA scaffolds and TCP on different time periods. The results are presented average value of n = 3.
