# Supplementary material for: Covalent Surface Functionalization of Bovine Serum Albumin to Magnesium Surface to Provide Robust Corrosion Inhibition and Enhance In Vitro Osteo-Inductivity
Source: Polymers (Basel). 2020 Feb 13;12(2):439. doi: 10.3390/polym12020439 (PMC7077681; doi:10.3390/polym12020439)
Supplement: Supplementary file 1 [file polymers-12-00439-s001.pdf]

Supplementary Materials

Covalent Surface Functionalization of Bovine Serum Albumin to Magnesium Surface to Provide Robust Corrosion Inhibition and Enhance in vitro Osteo-Inductivity

Seo Yeon Lee [1], Sita Shrestha [1], Bishnu Kumar Shrestha [1,2,*], Chan Hee Park [1,2,*] and Cheol Sang Kim [1,2,*]

[1] Department of Bionanosystem Engineering, Graduate School, Jeonbuk National University, Jeonju 561-756, Korea; sylee@jbnu.ac.kr (S.Y.L.); sitamhenpi123@gmail.com (S.S.)

[2] Division of Mechanical Design Engineering, Jeonbuk National University, Jeonju 561-756, Korea

* Correspondence: bishnuaamppipal@gmail.com (B.K.S.); biochan@jbnu.ac.kr (C.H.P.); chskim@jbnu.ac.kr (C.S.K.)

MTT assay test

Cell viability test was conducted to evaluate the cells proliferation, survivability and cytocompatibility of pre-osteoblast (MC3T3-E1) cells on the as-prepared scaffolds and were continually monitored up to 7-day period using MTT assay at interval of 1, 3 and 7-days of cell culture. MTT assay based on the ability of metabolically active cells to reduce MTT, a yellowish tetrazolium salt, to purple-colored formazan crystal by mitochondrial dehydrogenases. Briefly, at desired time point, the cell-seeded samples were washed with PBS and 100μL of a MTT (5mg/mL) labeling mixture was added to each well and incubated. After 4 h, the MTT containing medium was removed from each well plate and the insoluble formazan dissolved in 200 μL of dimethyl sulfoxide (DMSO). The optical density of the final solution was measured at a wavelength of 590 nm using an ELIZA reader.

Figure 1. shows the cell viability and proliferation on TCP and Mg–OH–AA–BSA scaffold at various time periods assessed by the MTT assay result. Cells were proliferated on both TCP and Mg–OH–AA–BSA, but not significant difference on day 1. However, at day (3rd and 7th) the cells were highly proliferated on the BSA modified Mg surface compared to TCP indicating the strong biocompatibility of Mg modified surface. The strong bonding of BSA on Mg modified surface exhibits bio-interface and possess cell to substrate interaction without any interference (for example fast corrosion of Mg). It is well known that the protein rich scaffolds are the best microenvironment for cellular metabolism and promising material for the application of implants in tissue engineering application, specially substitute material for auto/allografts. Thus, we confirm that the Mg–OH–AA–BSA scaffold supports our hypothesis with displaying anti-corrosion behavior and biocompatibility towards pre-osteoblast (MC3T3-E1) cells

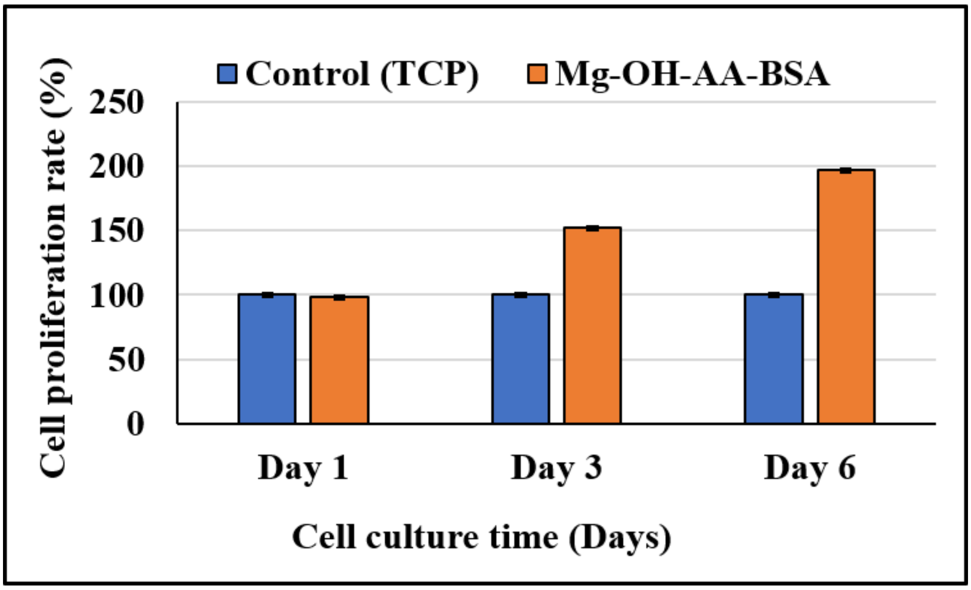

Figure S1. Cell growth and viability analysis using MTT assay to confirm the proliferation of pre-osteoblast (MC3T3-E1) cells on Mg–OH–AA–BSA scaffolds and TCP on different time periods. The results are presented average value of n=3.
